# Who Is Delirium Worse For?

**DOI:** 10.1111/jgs.70539

**Published:** 2026-06-15

**Authors:** Thiago J. Avelino-Silva, Márlon J. R. Aliberti, Mfon E. Umoh, Esther S. Oh

**Affiliations:** 1 Division of Geriatrics, Department of Medicine, University of California, San Francisco, California, USA; 2 Laboratório de Investigação Médica Em Envelhecimento (LIM-66), Serviço de Geriatria, Hospital das Clínicas HCFMUSP, Faculdade de Medicina, Universidade de São Paulo, São Paulo, Brazil; 3 Research Institute, Hospital Sírio-Libanês, São Paulo, Brazil; 4 Division of Geriatric Medicine and Gerontology, Department of Medicine, Johns Hopkins School of Medicine, Baltimore, Maryland, USA

**Keywords:** cognition, delirium, dementia, mortality, prognosis

Delirium is common in hospitalized older adults and is associated with mortality, institutionalization, cognitive decline, functional loss, and delayed recovery [1–12]. The question of whether the prognosis after delirium varies by baseline cognition is less often considered. As dementia reduces cognitive reserve and increases vulnerability to delirium, it is natural to assume that delirium superimposed on dementia would portend the worst prognosis. This assumption is reinforced by the clinical experience of patients with multiple conditions and vulnerabilities having worse outcomes than those with fewer issues. However, how common a condition is (prevalence) differs from how it will turn out (prognosis). For example, although delirium is more frequent in patients with dementia, evidence suggests that its association with mortality is strongest in patients without dementia [1, 3, 10, 13, 14]. This distinction is important when managing delirium.

“Although delirium is more frequent in patients with dementia, evidence suggests that its association with mortality is strongest in patients without dementia.”

More than two decades ago, McCusker and colleagues reported that delirium predicted 12-month mortality among older medical inpatients independent of dementia and other measured vulnerabilities [1]. Mounting evidence suggests that baseline cognition may modify the magnitude, timing, and clinical implications of that association. In a prospective cohort of 1409 hospitalized older adults, in-hospital mortality was 29% among patients with delirium alone, 32% among those with delirium superimposed on dementia, and 12% among those with dementia alone. After adjusting for confounders, delirium alone was associated with a higher risk of in-hospital death than delirium superimposed on dementia, while dementia alone was not independently associated with mortality [3]. In a Scottish cohort of 6724 patients, patients with delirium but no dementia had higher mortality immediately after admission. In contrast, among patients with known dementia, the excess mortality emerged only 3 months after admission [13]. Similar findings were reported in a 10-year observational study from England, in which delirium was more strongly associated with adverse outcomes in patients without dementia than in those with dementia [10]. This pattern has also been observed outside acute hospitals: in skilled nursing facilities, residents with delirium but no dementia had substantially higher mortality than those without delirium or dementia [14]. More recently, a multinational cohort study of 2556 hospitalized older adults found that delirium was associated with higher 90-day mortality across all levels of baseline cognition, from normal cognition (Clinical Dementia Rating [CDR] = 0) to moderate-to-severe dementia (CDR = 2–3) [15]. However, the increase in risk was much greater among patients without dementia (Figure 1): mortality rose from 15% to 54%, an absolute increase of 39%. Among those with moderate to severe dementia, mortality rose from 17% to 36%, a 19% increase.

Differences in cognitive reserve may partly explain this pattern. Patients with dementia are more susceptible to delirium, such that relatively modest physiologic stress may be sufficient to disrupt attention, arousal, and cognition. Conversely, patients with normal baseline cognition may require a more severe precipitating illness (e.g., sepsis instead of a mild infection, a significant metabolic imbalance rather than mild dehydration, or multisystem illness rather than transient physiologic stress) for delirium to develop [13]. The prognostic significance of delirium may therefore reflect not only baseline vulnerability but also the severity of the acute insult needed to trigger it.

This should not be taken to downplay delirium superimposed on dementia, which remains common and is associated with pro-longed hospitalization, complications, functional decline, and other adverse outcomes [16–18]. Notably, patients with dementia usually have higher baseline mortality than those without dementia and are more susceptible to hospital complications even before delirium occurs [3, 13]. Moreover, dementia is often undiagnosed at admission, and patients labeled “without dementia” may include those with unrecognized impairment [19]. But the more relevant clinical question is what delirium adds to that underlying risk. And, in this context, the excess risk associated with delirium appears greatest in patients without dementia or with milder cognitive impairment, suggesting that in these patients, delirium may indicate substantial clinical insult [3, 10, 13, 15]. Thus, the same syndrome may carry different prognostic implications depending on the baseline cognitive level at which it arises.

Longer-term outcomes are also critically important to consider. Patients who start with better baseline cognition may experience the greatest absolute decline once delirium develops. In a prospective, population-based study, individuals with high baseline cognitive performance who had a greater delirium burden demonstrated the largest decline in cognitive abilities [7]. This pattern suggests a loss of the usual protective effect associated with high premorbid cognition. Furthermore, after ≥ 2 days of moderate delirium, 2-year mortality rates were similar across cognitive levels, suggesting that delirium may diminish the survival advantage typically associated with better baseline cognition [7]. In the Oxford Vascular Study, delirium independently predicted later dementia after accounting for infection and white matter disease, with a particularly strong association among patients who were cognitively unimpaired at baseline [8].

UK Biobank data also support delirium as more than a transient hospital symptom, revealing its association with several subsequent adverse outcomes independent of frailty and pre-existing dementia [11]. Penfold and colleagues found that delirium after emergency admission was associated with incident dementia across levels of multimorbidity, with the strongest association in patients without multimorbidity [12]. Other studies in skilled nursing facilities point in the same direction, indicating residents with delirium without dementia can experience worse cognitive and functional recovery [6, 9]. These findings suggest that delirium is not merely a marker of acute illness severity but also a potential turning point in later health trajectories. For older adults who were previously cognitively healthy, the consequences of delirium may extend beyond recovery from the acute episode, potentially leading to a less favorable course of aging.

Several practical implications for clinical care follow. Delirium should not be approached primarily as a problem of dementia. Hospitals that concentrate detection and prevention efforts mainly on patients with known risk factors may miss the group in which delirium is least expected but may have the greatest prognostic significance. A more useful approach is to view delirium programs as hospital-wide patient safety efforts, with similar urgency whether delirium occurs in a patient with advanced dementia or in one with no documented cognitive diagnosis. This broader approach is also sensible because baseline dementia is often unrecognized or poorly documented at admission, and reliance on the medical record alone may obscure the clinical context in which delirium develops. Baseline cognition should be assessed early and deliberately, ideally with input from a family member or other informant, because the clinical implications of delirium depend in part on the patient’s premor-bid cognitive state. This information can improve prognostication, guide communication with families, and help differentiate new decline from longstanding impairment.

Furthermore, when delirium occurs in a patient presumed to be cognitively normal, clinicians should respond by escalating evaluation rather than offering reassurance. The response should include a renewed search for reversible precipitants, reassessment of illness severity, review of medications and iatrogenic contributors, attention to hydration, oxygenation, pain, sleep, mobility, sensory impairment, and closer monitoring. In this situation, delirium may be the sign that the current diagnosis or level of care is incomplete, and the possibility of increased mortality risk should not be overlooked. If a cognitively intact older adult develops delirium, one must carefully look for what changed enough to overwhelm their reserve.

Finally, discharge planning should recognize that delirium can remain important after the hospital stay. Patients and caregivers may expect the patient to return quickly to baseline, but that may not happen. Before discharge, clinicians should review medications, explain what symptoms to watch for, and arrange follow-up to check cognition and function. Families should know that ongoing inattention, poor sleep, hallucinations, loss of independence, or continued confusion should prompt reassessment. Recovery from delirium is not always complete or immediate; some patients improve only partly, and the outcomes may depend on their baseline cognition [2, 4, 17]. A discharge summary that simply says “delirium resolved” may miss ongoing risks, including new functional dependence, caregiver strain, and emerging cognitive impairment.

This perspective also has implications for research. Delirium research has rightfully focused on prevention in highly vulnerable populations, especially patients with dementia, and that work remains crucial. At the same time, the available evidence indicates a need to investigate whether earlier recognition and more aggressive management can lower mortality and cognitive decline in patients without known dementia, where delirium may be less expected but carries greater prognostic significance. Trials of detection, prevention, and postdischarge management in this population would be particularly valuable. Additionally, studies should categorize patients by baseline cognition, treating dementia as more than just a covariate, and offering a clearer understanding of how delirium risk and outcomes vary across different cognitive levels. Outcomes should include not only mortality and length of stay, but also cognition, function, institutionalization, caregiver burden, and quality of recovery.

The relationship between dementia, delirium, and mortality is more nuanced than we often assume. Dementia increases the risk of delirium. But when delirium occurs without dementia, it may be signaling a particularly severe clinical problem. That should serve as a crucial message about prognosis, triage, and urgency at the bedside. In other words, delirium should always command attention. In older adults without dementia, it may warrant even more.

## Figures and Tables

**FIGURE 1 |:**
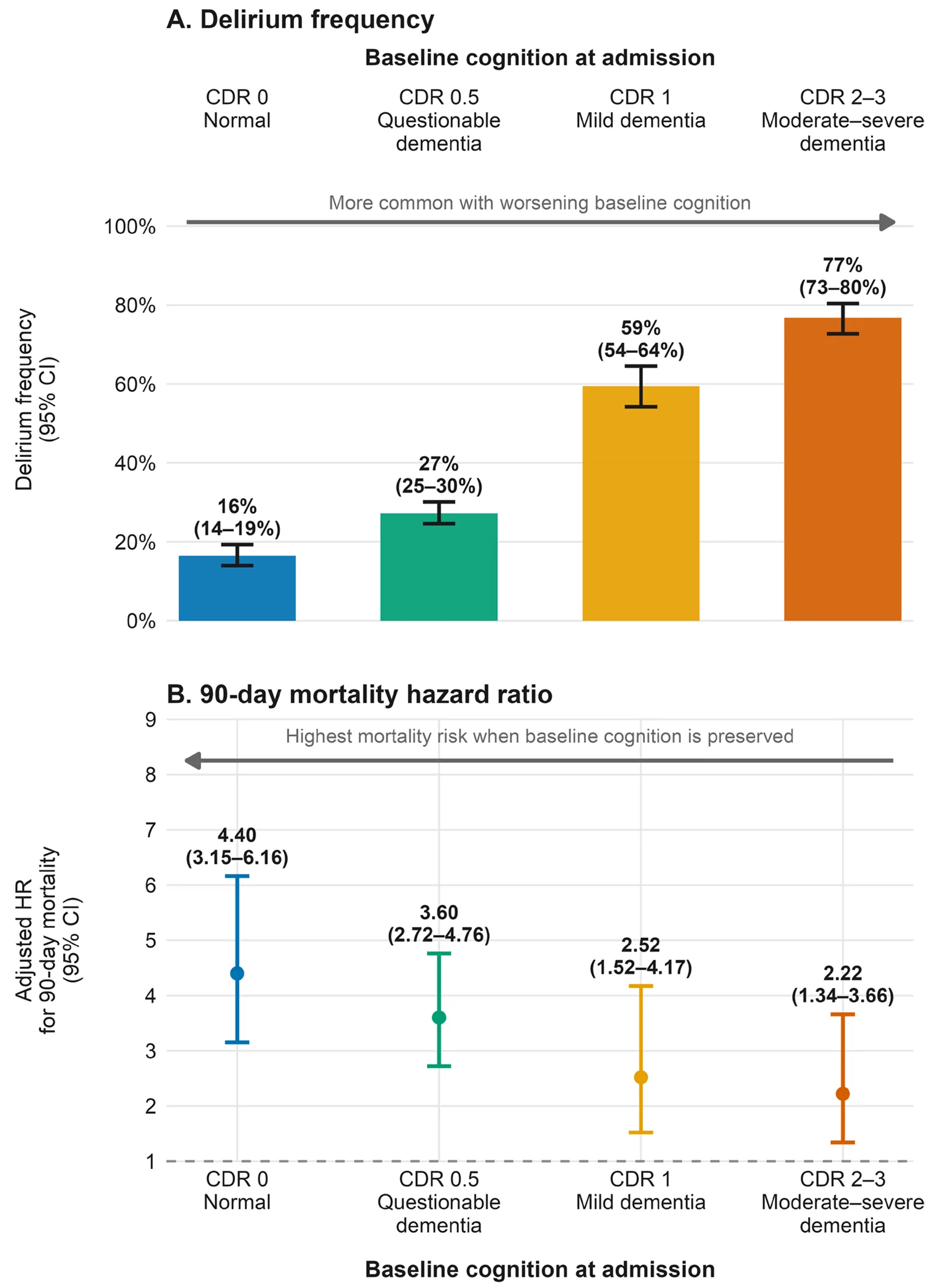
Delirium frequency and 90-day mortality risk by baseline cognition in hospitalized older adults. Panel A shows the frequency of delirium among hospitalized older adults across baseline cognitive strata, defined by CDR category. Panel B shows adjusted hazard ratios for 90-day mortality associated with delirium versus no delirium within each CDR category. Although delirium becomes more frequent as baseline cognition worsens, its association with 90-day mortality is strongest among patients without dementia. Error bars indicate 95% CIs. CDR, Clinical Dementia Rating; CI, confidence interval; HR, hazard ratio [15].
